# VisionMol: a novel virtual reality tool for protein molecular structure visualization and manipulation

**DOI:** 10.1093/bioinformatics/btaf118

**Published:** 2025-03-17

**Authors:** Xin Wang, Yicheng Zhuang, Wenrui Liang, Haoyang Wen, Zhencong Cai, Yujia He, Yuxi Su, Wei Qin, Yuanzhe Cai, Lixin Liang, Bingding Huang

**Affiliations:** College of Big Data and Internet, Shenzhen Technology University, Shenzhen 518118, China; College of Big Data and Internet, Shenzhen Technology University, Shenzhen 518118, China; College of Big Data and Internet, Shenzhen Technology University, Shenzhen 518118, China; College of Big Data and Internet, Shenzhen Technology University, Shenzhen 518118, China; College of Big Data and Internet, Shenzhen Technology University, Shenzhen 518118, China; College of Big Data and Internet, Shenzhen Technology University, Shenzhen 518118, China; College of Big Data and Internet, Shenzhen Technology University, Shenzhen 518118, China; College of Big Data and Internet, Shenzhen Technology University, Shenzhen 518118, China; College of Big Data and Internet, Shenzhen Technology University, Shenzhen 518118, China; College of Big Data and Internet, Shenzhen Technology University, Shenzhen 518118, China; College of Big Data and Internet, Shenzhen Technology University, Shenzhen 518118, China

## Abstract

**Motivation & Results:**

Virtual reality (VR) technology holds significant potential for applications in biomedicine, particularly in the visualization and manipulation of protein molecular structures. To facilitate the study of protein molecules and enable the state-of-the-art VR hardware, we developed a novel VR software named VisionMol, which allows users to engage in immersive exploration and analysis of 3D molecular structures using a range of VR platforms (such as Rhino X Pro, Meta’s Oculus Quest Pro/3) as well as personal computers. Built on the Unity engine and programmed using C#, VisionMol incorporates custom scripts to enable a variety of molecular operations. Users can rotate, scale, and translate molecular models using gestures, controllers, or other input devices. Furthermore, VisionMol offers rich visualization and interactive features, including multi-model molecular display, distance measurement between molecular components, and molecular alignment and docking.

**Summary:**

These capabilities facilitate a more intuitive understanding of molecular interactions and chemical properties. The real-time interactive effects and clear visual representations allow users to delve deeper into the relationships between molecular structures and their properties, thereby accelerating research progress and promoting scientific discovery. We believe that this VR-based protein molecule analysis has significant application value in several fields, including biomedicine, life science education, drug design and optimization, biotechnology, and engineering applications.

**Availability and implementation:**

The code is at https://github.com/WangLabforComputationalBiology/VisionMol. The v1.1 code (for Oculus Quest) could also be found at https://doi.org/10.5281/zenodo.14705790. The v1.0 code (for Rhino X Pro) could also be found at https://doi.org/10.5281/zenodo.14865216. Detailed documentation could be found at https://visionmol.surge.sh/#/en-us/README.

## Graphical Abstract

**Graphical Abstract:**
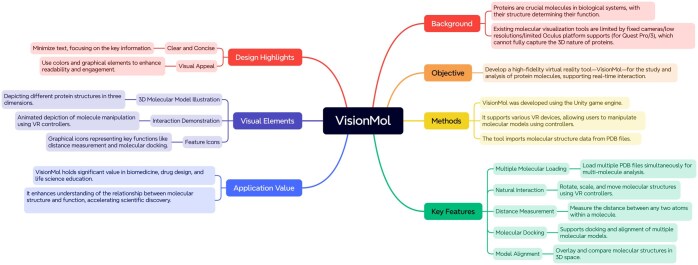

## 1 Introduction

Proteins are crucial molecular entities within biological systems, with their structure dictating their function. The scientific community has relentlessly pursued the study of protein molecules. By delving into the intricacies of protein molecules, we can gain insights into their secondary structures (such as α-helices and β-sheets) (Chakrabarti 2023) and tertiary structures (the 3D conformation of proteins) (Wuyun *et al.* 2024), thereby inferring potential functional mechanisms, including enzymatic activity, ligand binding, and signal transduction. In this process, protein molecule visualization technologies have emerged as a pivotal tool for analyzing protein structure and function. Currently available software like PyMOL (Schrödinger LLC 2015), UCSF Chimera (Pettersen *et al.* 2004), and Visual Molecular Dynamics (VMD) (Humphrey *et al.* 1996) offer extensive functionalities that enable users to effortlessly create molecular images and perform various structural analyses. Although these tools can display the 3D structure of proteins. However, these tools’ User experience (UX) is often limited by fixed cameras’ low resolutions, failing to fully convey the three-dimensionality of proteins in space. Moreover, user interactions typically rely on a mouse or keyboard, limiting interactivity to some extent.

Virtual reality (VR) technology provides a unique solution to address these issues. As a computer-generated simulation, VR allows users to immerse themselves and interact with a simulated environment, replicating sensory experiences of sight, sound, and touch, creating a sense of presence within the virtual world (Cruz-Neira *et al.* 1992). With recent advancements in VR technology, including the reduction in cost and improvement in performance of hardware, VR has become a burgeoning tool for molecular visualization.

As highlighted in various studies, VR visualization technology has been widely applied in biology. Currently, there are several 3D molecular visualization software developed based on VR technology, including (Holland *et al.* 2008), BioVR (Zhang *et al.* 2019), UnityMol (Doutreligne *et al.* 2014), and Molecular Rift (Norrby *et al.* 2015). In comparison to traditional molecular visualization tools like Pymol, these VR-based molecular visualization software offer distinct advantages. They provide immersive experiences, aiding users in better understanding and analyzing molecular structures. However, there are some limitations due to the nascent stage of VR technology when these software solutions were launched between 2016 and 2019. High costs associated with VR hardware and limited accessibility. Furthermore, despite providing immersive 3D molecular visualization, the available molecular models and data are relatively limited, which may not meet the specific needs of certain research areas. Moreover, the technological environment at the time also limited the stability and complexity of these software solutions.

To address these challenges, we have developed a new multi-functional VR molecular visualization tool called “VisionMol.” Upon entering the PDB ID (wwPDB Consortium 2019) of a protein molecule, VisionMol displays its 3D structure, which users can manipulate and analyze using VR controllers and other hardware devices.

## 2 Materials and methods

We selected Unity3D (hereafter referred to as Unity) as the development platform for VisionMol. Unity is a cross-platform game engine and application development tool, widely recognized as one of the most popular game development engines globally. Beyond gaming, Unity is extensively used in VR, augmented reality, 3D modeling, animation production, and engineering visualization. Therefore, Unity was the chosen platform for developing VisionMol.

### 2.1 Molecular data handling: PDB file format and parser

The Protein Data Bank (PDB) file format is used to store macromolecular structural data, primarily recording the 3D structures of proteins, nucleic acids, and other biomolecules (wwPDB Consortium 2019). VisionMol adopts UnityMol’s model import method and includes a built-in PDB file parser that effectively reads and interprets the 3D structural information of protein molecules from PDB files. Subsequently, utilizing Unity’s rendering technology, VisionMol transforms this information into 3D molecular structures displayed within the VR space.

### 2.2 Game objects and component-based programming in VisionMol

In Unity, each model is referred to as a “game object.” A complete protein molecule can be viewed as one game object, with each atom also represented as individual game objects. Components are fundamental parts of Unity game objects, enabling the functionality and behaviors. Unity allows users to script components on objects to implement custom functionality. According to official documentation, script components in Unity are primarily written in C#, the predominant programming language within the Unity ecosystem. In VisionMol, we predominantly utilize C# to script components that add various functionalities to the protein molecule models. Through these custom script components, we have implemented several functions, such as calculating distances between atoms within a single molecule, displaying various atomic labels, and comparing alignments among multiple molecules.

### 2.3 VisionMol’s user interface and hardware compatibility

**Figure 1.**
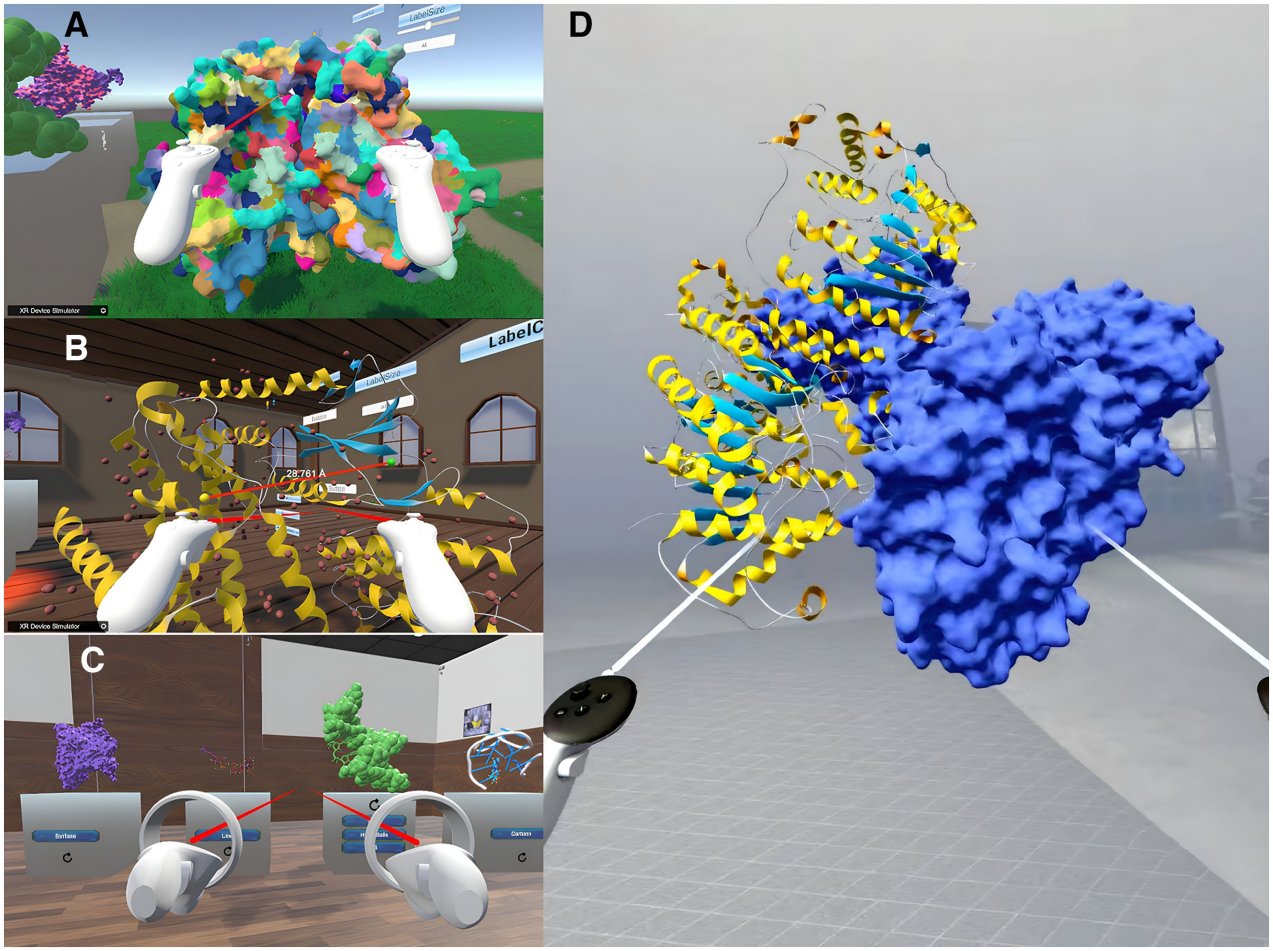
The illustration of VisionMol. (A) Illustrates the main interface of VisionMol, where users can independently select the molecular display models. (B) Showcases the molecular distance measurement feature of VisionMol. (C) Depicts the functionality for color coding different regions of molecules. (D) Demonstrates the molecular docking capabilities of VisionMol.

VisionMol’s interface is a virtual scene built with Unity, as shown in Fig. 1A. Additionally, to accommodate different user preferences, VisionMol also supports simulation of various VR hardware operations using a computer screen and mouse, allowing it to run on users’ personal computers.

## 3 Key features

VisionMol’s capability to present and manipulate 3D molecular structures within one VR environment holds significant scientific, medical, and educational implications. Through VisionMol, users can explore and understand the 3D molecular world in unprecedented ways. We firmly believe that VisionMol will drive progress across various disciplines in the future.

This study developed a VR tool called VisionMol, designed to visualize 3D molecular structures within a VR environment while offering interactive manipulation capabilities. The key features provided by VisionMol include the following:

1. The ability to load multiple molecular files in PDB format simultaneously within the VR scene.
2. Natural 3D interaction using VR controllers, enabling intuitive translation, rotation, and scaling of the molecular structures.
3. Support for various classical molecular representation methods, such as wireframe, stick, ball-and-stick, surface, and protein cartoon models.
4. Measurement of the distance between any two atoms within a molecule.
5. The ability to merge multiple molecular models into a unified composite structure.
6. Display and hide atomic labels within the 3D molecular models.
7. Visualization of molecular models divided into distinct color blocks based on residue grouping.
8. Customization of color schemes for protein secondary structures, including alpha-helices and beta-sheets.

Traditional 2D representations, such as static images and 3D renderings on a flat screen, fail to accurately convey the spatial arrangement and dynamic behaviors of molecules (National Research Council 2006). However, through these features, VisionMol enables a precise representation of the spatial arrangement and dynamic structure of molecules, allowing users to conduct high-fidelity analyses of 3D models within a virtual environment, thereby significantly enhancing analytical efficiency.

Additionally, VisionMol is compatible not only with VR platforms like Oculus and Rhino X, but also with personal computers, providing flexibility for users with different hardware setups. VisionMol’s unique features, including high-resolution, interactive 3D molecular visualization, offer substantial value to researchers working with complex molecules. For laboratories without VR equipment, we are exploring options to adapt some of these features to standard 3D monitors, ensuring broader accessibility.

Furthermore, while traditional 3D protein visualization software meets basic scientific needs, VisionMol aims to expand its application, particularly in education and training. VR’s intuitive and interactive capabilities provide a more engaging learning experience for students and educators.

In the experiments, we tested VisionMol’s functionalities using various molecular models. For instance, the molecular distance measurement feature was evaluated with the molecule corresponding to PDB ID 6P8E, with the results shown in Fig. 1B. Similarly, using the 6P8E molecule, we tested the functionality of dividing the model into distinct color blocks based on residues, with the final model depicted in Fig. 1C. We also tested the secondary structure color adjustment feature in VisionMol. During this process, a 3D color palette is displayed, allowing users to manipulate the RGB values to individually adjust the colors of the various structural levels within the molecular model. Additionally, the molecular docking capability of VisionMol was tested using molecules 6WBW and 7ZZO. In this test, the model of 7ZZO was rendered using the surface representation, while 6WBW remained in the default cartoon representation, as shown in Fig. 1D.

## 4 Discussion

VisionMol enhances the analysis of multiple protein molecules by allowing users to import and customize both identical and distinct models, facilitating simultaneous analyses that reveal structural similarities, such as those between hemoglobin and myoglobin, crucial for oxygen transport (Perutz *et al.* 1960), and G protein-coupled receptors (GPCRs) with shared features (Stevens *et al.* 2013). It also supports measuring interatomic distances, displayed in angstroms (Å), which is vital for assessing distances between protein atoms in studies (Borbat *et al.* 2002), evaluating binding efficacy in drug design (Morris *et al.* 2009), and determining coordination modes in coordination compounds (Blaney and Scott Dixon 1994). Additionally, VisionMol enables the merging and docking of molecular models, enhancing visualization of spatial relationships and allowing comparative analyses that inform potential functions and mechanisms. This capability is particularly valuable for simulating complex biological and chemical systems, advancing the understanding of their structures and regulatory mechanisms (Guo *et al.* 2008). VisionMol also supports the alignment of multiple molecular models by allowing users to customize collision volumes for enhanced spatial overlap, which is crucial for structural comparison in drug design and quantitative structure–activity relationship (QSAR) studies (Gramatica 2007).

Regarding the performance characteristics of VisionMol, our benchmarking tests with standard PDB datasets (e.g. 1HHO [hemoglobin], 1TIM [triose phosphate isomerase]) demonstrate consistent frame rates over 90 FPS on mid-range GPUs (GTX 1080 equivalent). However, we observed frame rate fluctuations (45–60 FPS) when handling exceptionally large complexes like 1KF1 (ribosome, about 2.5 million Daltons) and 6VW1 (COVID-19 spike glycoprotein complex). This aligns with industry benchmarks; for comparison, PyMOL requires 12–18 s for initial 1KF1 rendering on equivalent hardware, while VisionMol averages 9–15 s.

Regarding hardware requirements, we formalized a three-tier specification guideline:

- Minimum: GTX 1060 (4 GB VRAM), 4-core CPU, 8 GB RAM (30 FPS @ 1M Daltons)
- Recommended: RTX 3060 (8 GB VRAM), 6-core CPU, 16 GB RAM (60 FPS @ 3M Daltons)
- Professional: RTX 4090 (24 GB VRAM), Threadripper CPU, 32 GB RAM (90+ FPS @ 5M Daltons)

In order to better highlight the effectiveness of VisionMol, we have created a functional comparison chart with most similar products on the market, emphasizing the differences in VR platform applications (Supplementary Table S1).

In future, we will develop an integrated performance diagnostic tool that will automatically suggest rendering quality presets based on detected hardware capabilities. Also, we will explore out-of-core rendering for structures exceeding GPU memory, WebAssembly port for cloud-based preprocessing and machine learning-based topology prediction to bypass exhaustive verification.

## Supplementary Material

btaf118_Supplementary_Data

## Data Availability

The v1.1 code (for Oculus Quest) could be found at https://doi.org/10.5281/zenodo.14705790. The v1.0 code (for Rhino X Pro) could be found at https://doi.org/10.5281/zenodo.14865216. Detailed documentation could be found at https://visionmol.surge.sh/#/en-us/README.
