# Supplementary material for: VisionMol: a novel virtual reality tool for protein molecular structure visualization and manipulation
Source: Bioinformatics. 2025 Mar 17;41(3):btaf118. doi: 10.1093/bioinformatics/btaf118 (PMC11947413; doi:10.1093/bioinformatics/btaf118)
Supplement: btaf118_Supplementary_Data [file btaf118_supplementary_data.pdf]

Supplementary Table 1: Comparison of VisionMol and other similar software.

Feature/Function	VisionMol	PyMOL	Chimera	VMD	3Dmol.js	Jmol
**Operating Platform**						
- Android (VR Devices)	✓	✗	✗	✗	✗	✗
- Windows (Desktop)	✓	✓	✓	✓	✗	✓
- macOS (Desktop)	✓	✓	✓	✓	✗	✓
- Linux (Desktop)	✓	✓	✓	✓	✗	✓
- Web Browser	✗	✗	✗	✗	✓	✓
**Protein Molecule Loading**	✓	✓	✓	✓	✓	✓
**Molecular Representation**						
- Cartoon	✓	✓	✓	✓	✓	✓
- Line	✓	✓	✓	✓	✓	✓
- Surface	✓	✓	✓	✓	✓	✓
- VDW	✓	✓	✓	✓	✓	✓
- Hyperball	✓	✓	✓	✗	✗	✗
**Molecular Docking**	✓	✓	✓	✓	✗	✗
**Molecular Distance Measurement**	✓	✓	✓	✓	✓	✓
**Molecular Labels**						
- Residues	✓	✓	✓	✓	✓	✓
- Chains	✓	✓	✓	✓	✓	✓
- Atom Name	✓	✓	✓	✓	✓	✓
- Element Symbol	✓	✓	✓	✓	✓	✓
- Residue Name	✓	✓	✓	✓	✓	✓
- B-factor	✓	✓	✓	✓	✗	✗
- Vdw	✓	✓	✓	✓	✓	✓
**Controller/Hand Gesture**	✓	✗	✗	✗	✗	✗
**Immersive Experience**	✓	✗	✗	✗	✗	✗
